# Ethical, legal, and social aspects of symptom checker applications: a scoping review

**DOI:** 10.1007/s11019-022-10114-y

**Published:** 2022-10-01

**Authors:** Regina Müller, Malte Klemmt, Hans-Jörg Ehni, Tanja Henking, Angelina Kuhnmünch, Christine Preiser, Roland Koch, Robert Ranisch

**Affiliations:** 1 Institute of Ethics and History of Medicine, University of Tübingen, Gartenstraße 47, 72074 Tübingen, Germany; 2 Institute of Applied Social Sciences, University of Applied Sciences Würzburg-Schweinfurt, Münzstraße 12, 97070 Würzburg, Germany; 3 Institute of Occupational and Social Medicine and Health Services Research, University Hospital Tübingen, Wilhelmstraße 27, 72074 Tübingen, Germany; 4 Institute for General Practice and Interprofessional Care, University Medicine Tübingen, Osianderstraße 5, 72076 Tübingen, Germany; 5 Faculty of Health Sciences Brandenburg, University of Potsdam, Karl-Liebknecht-Str. 24-25, House 16, 14476 Potsdam, Golm, Germany

**Keywords:** Digitalisation, mHealth, Health apps, Symptom checker apps, Bioethics, Scoping review

## Abstract

Symptom Checker Applications (SCA) are mobile applications often designed for the end-user to assist with symptom assessment and self-triage. SCA are meant to provide the user with easily accessible information about their own health conditions. However, SCA raise questions regarding ethical, legal, and social aspects (ELSA), for example, regarding fair access to this new technology. The aim of this scoping review is to identify the ELSA of SCA in the scientific literature. A scoping review was conducted to identify the ELSA of SCA. Ten databases (e.g., Web of Science and PubMed) were used. Studies on SCA that address ELSA, written in English or German, were included in the review. The ELSA of SCA were extracted and synthesized using qualitative content analysis. A total of 25,061 references were identified, of which 39 were included in the analysis. The identified aspects were allotted to three main categories: (1) Technology; (2) Individual Level; and (3) Healthcare system. The results show that there are controversial debates in the literature on the ethical and social challenges of SCA usage. Furthermore, the debates are characterised by a lack of a specific legal perspective and empirical data. The review provides an overview on the spectrum of ELSA regarding SCA. It offers guidance to stakeholders in the healthcare system, for example, patients, healthcare professionals, and insurance providers and could be used in future empirical research to investigate the perspectives of those affected, such as users.

**Supplementary Information:**

The online version contains supplementary material available at 10.1007/s11019-022-10114-y.

## Background

Digital health technologies have grown immensely in the health sector over the last decades. This development is also present in the field of so-called mobile Health (mHealth) and might have the potential to change health care, health research, and the education of health practitioners significantly (Mohapatra et al. 2015; Ali et al. 2016; Marcolino et al. 2018). Mobile devices, in particular smartphones, have become indispensable for the daily activities of many people. By 2021, about 85% of the US population owned a smartphone (Pew Research Center 2021) and the mobile-health market has grown steadily in recent years, with mobile-health apps already becoming an integral part of our lifestyle and healthcare (Research 2 Guidance 2017). These health apps cover a wide range of functions, for example, for communication, access to health information, or for monitoring health while addressing different user groups, such as healthy individuals, patients, or healthcare professionals (Mosa et al. 2012; Carroll et al. 2017).

Promising and widely used health apps that have gained traction in recent years include symptom checker apps (SCA). SCA are digital applications, often for mobile devices, to assist with symptom assessment and/or self-triage.^1^ These commonly chatbot-based apps promise diagnostic help insofar as the apps propose a list of potential causes for symptoms entered into the app, usually ordered by likelihood of occurrence. These results inform the user about possible conditions that could explain their symptoms. In addition, the triage function gives recommendations on whether to seek help, and if so, where (e.g., at the general practitioner’s or emergency department), and indicating the degree of urgency (e.g., within a few days). Through these two main functions, SCA are intended to help with the assessment of symptoms and assignment to appropriate care.

Most SCA are freely available apps which have been developed for use on mobile devices such as smartphones or tablets. Current SCA include Ada, Symptomate, Your.MD, WebMD: Symptom Checker, and others. Although app-based and web-based symptom checkers can have similar functions, the aim of this review is to investigate the mobile use of SCA. Nowadays, internet use often occurs via mobile devices. Globally, minutes on the mobile continue to represent a majority of the time spent online. And of the time spent on mobile devices, apps take up the majority (Comscore 2020). Compared to websites, apps can also utilise typical functions of mobile phone hardware such as location services (GPS) or cameras and may even instantly link with other wearable devices. Notably, it can be assumed that the ubiquitous and pervasive nature of mobile technologies is associated with a different user behaviour, which may reveal specific ethical, legal, and social aspects (ELSA). Mobile phones can be carried on the body, are always on and always close at hand, allow instant and easy use, and many people have a special attachment to their devices, which often stores a whole range of personal (health-related) data (Klasnja and Pratt 2012). These unique qualities of mobile devices and mobile applications call for a distinct ELSA analysis.

Some health institutions, such as the UK National Health Service (National Health Service 2021) and the government of Australia (Healthdirect Australia 2021), have already adopted SCA. The apps advertise improvements on different levels of healthcare: End-users receive information about medical symptoms and guidance through the healthcare system. This information supposedly helps patients in seeking further medical advice on the appropriate level, in turn, reducing the workload and costs in the healthcare system. Whether or not these promises are kept and how such expectations affect consumers, health-care professionals, and the healthcare system remain to be investigated. However, the potential use of SCA already raises ethical, legal, and social aspects, such as the fair access to this technology, their impact on the physician-patient-relationship, and the moral and legal responsibility in the case of the incorrect assessment of symptoms.

Previous studies have focused on the quality and accuracy of the apps’ diagnosis and triage advice (Razzaki et al. 2018; Hill et al. 2020; Gilbert et al. 2020), their design and usability (Copeland et al. 2018), and usage characteristics (Morse et al. 2020; Arellano et al. 2022). Some reviews already exist about the use and performance of digital and online applications for diagnosis (Millenson et al. 2018; Aboueid et al. 2019; Chambers et al. 2019a; Wattanapisit et al. 2020) as well as several empirical studies about users’ perceptions of SCA, such as by patients (Verzantvoort et al. 2018; Meyer et al. 2020; Miller et al. 2020), young users (Aboueid et al. 2021), and healthcare professionals (Fiske et al. 2020; Kujala et al. 2020). Since many publications focus on specific health conditions, for example, on ophthalmic diagnoses (Shen et al. 2019), HIV, and hepatitis C (Berry et al. 2019), the current review sets out to identify the ELSA of consumer-targeted SCA in a comprehensive and systematized way. It is embedded in the multi-disciplinary project CHECK.APP. In this collaborative project, we aim to gain empirical data about SCA in Germany from the perspectives of users/patients, general practitioners, and various experts of the healthcare system (Wetzel et al. 2022). The scoping review aims to address the following empirical question: Which ELSA occur in the scientific literature on SCA? We will provide an overview of a range of corresponding issues discussed in the literature.

## Methods

Due to the exploratory character of the research question, a scoping review methodology was chosen to review the literature on ELSA in the context of SCA. A scoping review encompasses the mapping of pertinent knowledge in the literature without evaluating the quality of empirical evidence. This seems relevant as, after an initial review of the literature on the current state of SCA, empirical evidence appears to be sparse and research designs are heterogeneous (Peters et al. 2015). In addition, a scoping review allows for normative literature to be collected alongside empirical works. The review method was guided by the methodological framework for scoping reviews PRISMA (Tricco et al. 2018) and reported accordingly (Suppl. 1). A review protocol was written and used.

The review included the following steps: (1) defining the research question, (2) identifying relevant studies, (3) selecting the studies, (4) charting the data, and (5) collating, summarizing, and reporting results. The ELSA are qualitatively extracted from the publications, presented in a descriptive manner, and discussed. The corresponding author of this study can provide the review protocol upon request.

### Eligibility criteria

The eligibility criterion for a publication to be included in the review was the mention of an ethical, legal, and/or social aspect related to SCA. However, there is no consensus in the literature of what exactly constitutes an ethical, legal, or social aspect, and explicit definitions, e.g. of common terms such as ‘ethical aspects’ or ‘ethical challenge’, vary (Schofield et al. 2021). No specific definition of an ethical, legal, or social aspect was used in this review so as not to limit the diversity of aspects identified. Rather, a broad understanding of ELSA was applied, drawing on the reviewers’ training and research experience in bioethics, jurisprudence, and social sciences. All terms (and their synonyms) that were found in the literature were considered. The aspects identified could be negative (problems, concerns, challenges, etc.), neutral (impacts, effects, etc.), or positive (benefits, opportunities, etc.). This review collects both manifest and anticipated aspects of SCA. In order to display the full spectrum of ELSA relevant to the review question, not only argument-based but also empirical literature (when discussing ELSA) was included.

SCA were defined as digital applications on mobile devices that support the assessment of symptoms and/or self-triage. In principle, SCA can be developed for very different platforms and even before the advent of smartphones there were web-based symptom checkers. Today, tools for symptom assessment are often app-based and made for use on mobile devices. Some services such as Symptomate or WebMD offer not only a (native) app (e.g. for iOS and/or Android) but also web applications that can be accessed through the web browser (e.g. on desktop computers or mobile devices). The literature dealing with SCA often does not distinguish between different types or platforms for such applications. In cases of ambiguity or cross-platform apps, we included the literature in our review, but focused in the analysis on the app-based versions for mobile devices, or rather on the characteristics of their mobile use.

While some SCA are made for the use by lay people and/or for the use by experts, applications specifically developed as support tools for health professionals or other experts were not included in the review. Since this review is intended to provide a comprehensive overview, only SCA that allow for a general symptom analysis were included. Applications, which focus on a specific health-condition only (e.g. diabetes) or certain groups of diseases (e.g. mental disorders), were excluded because these are associated with specific challenges. For example, in the context of mental-health issues such as anxiety or depression, patients or service users might be seen as especially vulnerable. Symptom checkers for these patient/user groups might be investigated with a focus on stigma or epistemic injustice. Another example are symptom checkers in the context of sexual health. Here, a sense of shame, gender aspects, or intersectional perspectives might be brought into focus. However, this does not mean that the included SCA cannot also cover these conditions among others.

Publications on SCA that were written in English or German and published up to 2020 were included in the review. Journal articles, contributions to anthologies, reports, case series, letters to editors, opinions, commentaries, and conference papers were included while online blogs were excluded.

### Search strategy and selection of studies

**Fig. 1:**
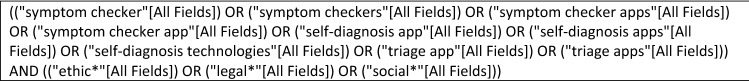
Search string (example PubMed)

Ten online databases relevant to the disciplines of interest (e.g., medicine, bioethics, law, and social sciences) were selected for the study: Web of Science, PubMed, Cochrane Library, Belit/Ethmed, ProQuest, Gesis/SowiPort, Philpapers, Juris, BeckOnline, and Google Scholar. Additionally, library catalogues and relevant journals were reviewed manually. Search terms were identified, tested through a preliminary explorative search, discussed among the research team and extended accordingly. Thereafter, RM, MK, and AK used the selected search terms for the search across all selected databases (search period: July to December 2020). The search strategy for the database PubMed can be seen in Fig. 1 as an example. These terms were altered to correspondingly specific terms used in other databases.

**Fig. 2:**
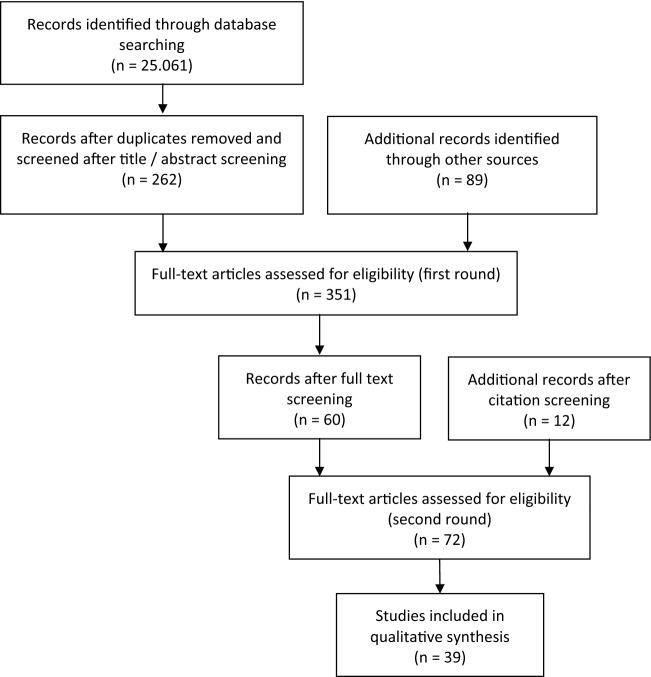
Selection of studies (flow diagram). (Moher et al. 2009)

RM, MK, and AK reviewed the identified literature (n = 25,061) by title and the screening of abstracts, resulting in n = 262 sources. To identify additional sources, a manual search in relevant journals (e.g., BMC Medical Informatics and Decision Making; American Journal of Public Health; Journal of Health Communication; Social Science & Medicine) was added, resulting in n = 89 additional publications. A database was created with the citation manager EndNote, including n = 351 publications. These publications were then randomly assigned to the three reviewers, who screened the full text of the selected literature and discarded publications clearly not meeting the inclusion criteria (first round). This first full-text screening resulted in a sample (n = 60) that still contained differing evaluations of some publications among the reviewers. When in doubt, publications were included ‘under reserve’. In the meantime, an additional screening of the references cited in the included literature (n = 60) was conducted. This resulted in n = 12 additional publications. A second screening of the remaining publications in full text (second round; n = 72), accompanied by repeated discussions between RM, MK, and AK led to agreement on the selected studies (n = 39). The selection process of the studies can be seen in Fig. 2. No appraisal of the quality of the included studies was conducted in the present review because there are no established criteria on how to conduct such an appraisal of the quality of reviews of argument-based literature (Mertz 2019).

### Data extraction and qualitative synthesis

The ELSA of SCA were extracted and synthesized by RM and MK using qualitative content analysis according to Kuckartz (2016) and the software MAXQDA Standard 2020. Considering the aims of a scoping review, qualitative content analysis was the methodology selected because it provides a systematized way of reducing, synthesizing, and structuring a wide range of data to extract main topics and sub-topics (Julien 2008). Its central idea is to systematically develop a coding frame that outlines the relevant topics of the data material (Julien 2008). Our coding frame includes deductive and inductive categories. Table 1 shows an example of the process for developing the categories.

**Table 1:** Category development process (examples)

Quote	Code	Subcategory	Category	Topic
Several of the apps that we examined were used for promotional purposes that were not always readily apparent at first sight (Jutel and Lupton 2015)	Promotional purposes/intransparency	Intransparency	(In)Transparency	Technology
Some of the health professionals believed that the symptom checkers were a threat to their professional autonomy as they reduced their control over the patient process and professional decision making (Kujala et al. 2020)	Threat to professional autonomy/control	Issues of professional power and authority	Patient-physician relationship	Individual level
A principal benefit described by respondents was the possibility for digital self-care technologies to expand the reach of health care to hard-to-serve populations, possibly facilitating earlier disease detection (Fiske et al. 2020)	Expanding the reach of health care/hard-to-serve populations	Improved access/advantageous for specific groups	Justice	Healthcare system

To develop a shared preunderstanding of ELSA in the context of SCA the reviewers RM and MK familiarized themselves with the literature and discussed potential aspects within the research team, which has experience in biomedical ethics, jurisprudence, and the social sciences. In order to not limit the variety of aspects to identify, a broad understanding of what constitutes an ethical, legal, or social aspect was chosen. First, categories were generated to provide a general guideline for analysis. With the aid of these categories and according to the research question, RM and MK independently screened the publications for ELSA of SCA. Codes were assigned inductively to each occurring aspect and initially subsumed in separate ethical, legal, and social categories. After all publications had been analysed once, the derived ethical, legal, and social categories were discussed in team meetings to foster intercoder-reliability (Schreier 2012). Preliminary categories were revised and overlapping categories and subcategories were brought together in one final coding frame according to three main topics. The three topics were generated deductively and resulted from team discussions on the instrumental aspects of the technology, the individual level of intra- or interpersonal relationships, and the institutional level of social contexts. To further increase trustworthiness (Elo et al. 2014), preliminary versions as well as the final version of the coding frame were discussed in joint meetings including researchers from all CHECK.APP subprojects. All publications were analysed a second time with this final coding frame by RM and MK to ensure the assignment of the correct code from the revised coding frame. Publications were also analysed by type and year of publication, by the methods used, and by the authors’ disciplines.

## Results

**Fig. 3:**
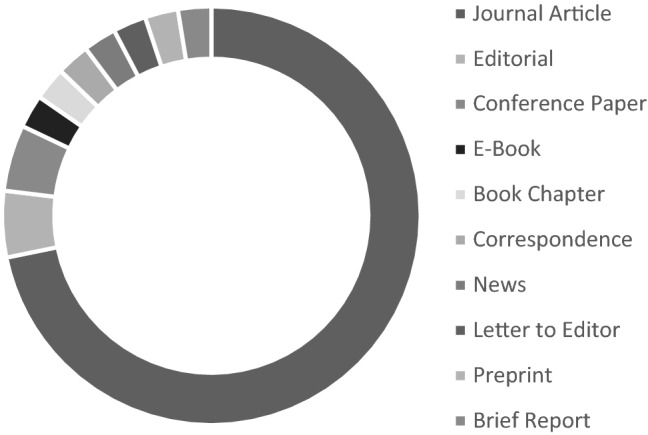
Types of publication. Publications (n = 39)

The search identified 25,061 articles, of which 39 publications were included in the analysis. The selection process of the studies can be seen in Fig. 2, the list of all included publications in Supplement 2. The selected studies are very heterogeneous in terms of publication type. They consist mainly of journal articles, but include various further types of work such as contributions to anthologies, editorials, or letters [Fig. 3].

**Fig. 4:**
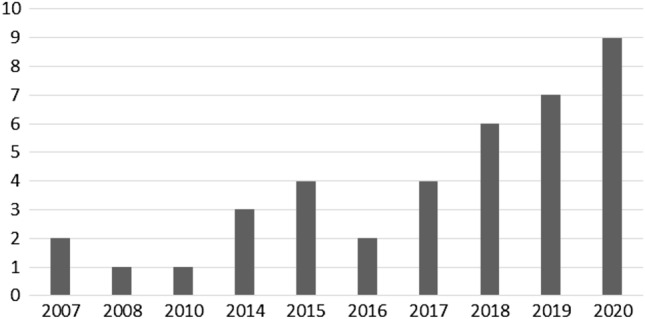
Publications per year. Publications (n = 39)

The vast majority of the articles reviewed were written in English (n = 33), some in German (n = 6). The included literature was published between 2007 and 2020. Figure 4 shows the publications per year, showing a gradual and clear rise of publications in this timespan.

**Fig. 5:**
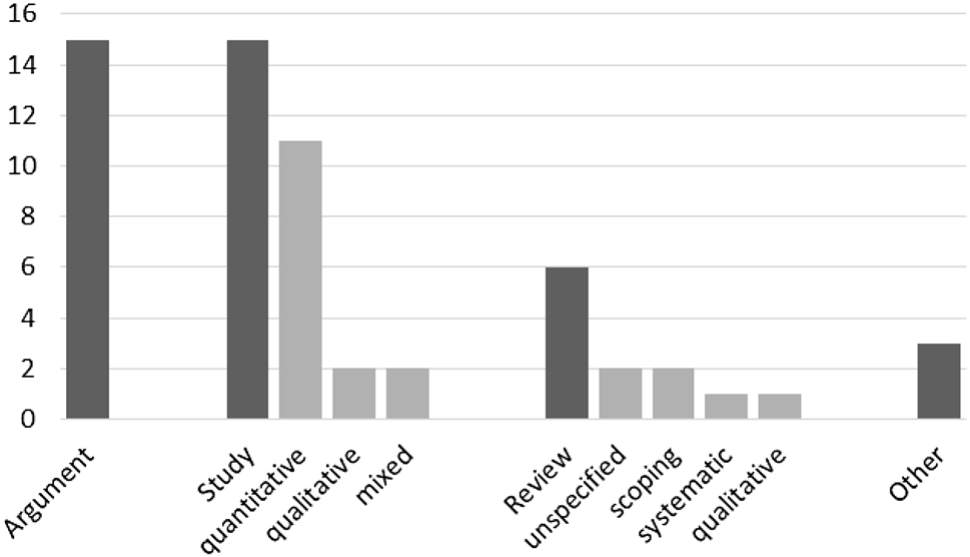
Methods. Publications (n = 39)

**Fig. 6:**
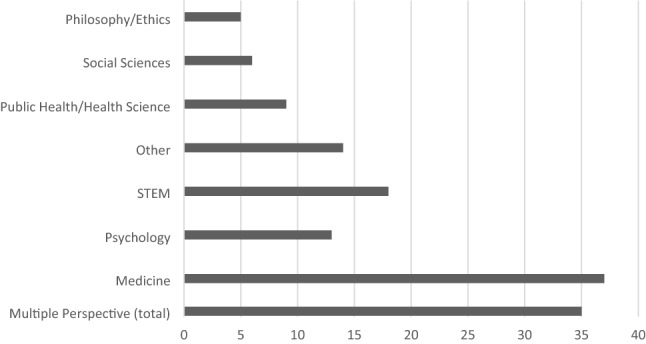
Authors’ disciplines. STEM fields: science, technology, engineering, mathematics

The publications cover different methods, for example, argument-based articles, empirical studies, reviews, and reports, with argument-based publications and empirical studies constituting the majority [Fig. 5]. Figure 6 shows the varied disciplines of the authors, most of them coming from medicine, a few from philosophy/ethics and other (inter-)disciplines. We noted each author's main discipline and if they have more than one discipline as background.

### Ethical, legal, and social aspects of SCA identified in the literature

The ethical, legal, and social aspects were categorized during the analysis by sorting them into three main categories: (1) aspects regarding the technology itself (*Technology*); (2) aspects concerning the individual level of intra- or interpersonal relationships (*Individual Level*); and (3) aspects at the institutional level of the healthcare system (*Healthcare System*). In the categorisation process, up to three levels of subcategories were identified and 78 codes were assigned all up. Table 2 shows all categories and subcategories, the frequency of their overall occurrence, and the publications in which a subcategory occurred at least once.

**Table 2:** Occurrence of categories and subcategories

Categories and subcategories	Frequency^a^	References
**Technology**		
Commercial aspects	10	Fiske et al. (2020), Dunn (2020), Fraser et al. (2018), Jutel and Lupton (2015), Lupton and Jutel (2015), Kramer (2017), Merz et al. (2018), Powley et al. (2016), Ryan and Wilson (2008), Sönnichsen (2019)
Expertise of producers/developers	5	Hill et al. (2020), Verzantvoort et al. (2018), Jutel and Lupton (2015), Lupton and Jutel (2015), Morita et al. (2017)
Lack of evidence	16	Millenson et al. (2018), Aboueid et al. (2019), Chambers et al. (2019), Kujala et al. (2020), Dunn (2020), Fraser et al. (2018), Kramer (2017), Merz et al. (2018), Ryan and Wilson (2008), Sönnichsen (2019), Hageman et al. (2015), Kao and Liebovitz (2017), Kuhn et al. (2018), Iacobucci (2020), Semigran et al. (2015), Wyatt (2015)
Lack of regulation (accuracy, quality, performance)	5	Fiske et al. (2020), Dunn (2020), Fraser et al. (2018), Kao and Liebovitz (2017), Iacobucci (2020)
(In)Transparency	7	Hill et al. (2020), Aboueid et al. (2019), Lupton and Jutel (2015), Kramer (2017); Sönnichsen (2019), Herzog (2019), Lanseng and Andreassen (2007)
Quality of the apps	6	Millenson et al. (2018), Fiske et al. (2020), Fraser et al. (2018), Jutel and Lupton (2015), Lupton and Jutel (2015), Ryan and Wilson (2008)
User participation in development	4	Aboueid et al. (2019), Kujala et al. (2020), Kao and Liebovitz (2017), Lanseng and Andreassen (2007)
**Individual level**		
Healthcare professionals	13	Hill et al. (2020), Razzaki et al. (2018), Wattanapisit et al. (2020), Aboueid et al. (2019), Fiske et al. (2020), Kujala et al. (2020), Dunn (2020), Lupton and Jutel (2015), Kramer (2017), Merz et al. (2018), Sönnichsen (2019), Kuhn et al. (2018), Semigran et al. (2015)
Diagnosis and guidance	10	Hill et al. (2020), Razzaki et al. (2018), Wattanapisit et al. (2020), Aboueid et al. (2019), Fiske et al. (2020), Dunn (2020), Lupton and Jutel (2015), Merz et al. (2018), Sönnichsen (2019), Kuhn et al. (2018)
Digital expertise	4	Kujala et al. (2020), Merz et al. (2018), Kuhn et al. (2018), Semigran et al. (2015)
Professional expertise	4	Hill et al. (2020), Fiske et al. (2020), Dunn (2020), Merz et al. (2018)
Responsibilities	2	Kujala et al. (2020), Kramer (2017)
User/patient-physician relationship	19	Hill et al. (2020), Razzaki et al. (2018), Wattanapisit et al. (2020), Aboueid et al. (2019), Meyer et al. (2020), Miller et al. (2020), Fiske et al. (2020), Kujala et al. (2020), Jutel and Lupton (2015), Lupton and Jutel (2015), Merz et al. (2018), Powley et al. (2016), Sönnichsen (2019), Morita et al. (2017), Kuhn et al. (2018), Semigran et al. (2015), Wyatt (2015), Thielscher and Antes (2019)
Characteristics of the relationship	4	Meyer et al. (2020), Miller et al. (2020), Fiske et al. (2020), Wyatt (2015)
Expectations	2	Meyer et al. (2020), Fiske et al. (2020)
Face-to-face-interaction	3	Miller et al. (2020), Fiske et al. (2020), Wyatt (2015)
Trust	1	Meyer et al. (2020)
Effects on the relationship	5	Hill et al. (2020), Aboueid et al. (2019), Fiske et al. (2020), Jutel and Lupton (2015), Lupton and Jutel (2015)
Procedures	3	Fiske et al. (2020), Merz et al. (2018), Kuhn et al. (2018)
Power	17	Hill et al. (2020), Razzaki et al. (2018), Wattanapisit et al. (2020), Aboueid et al. (2019), Meyer et al. (2020), Miller et al. (2020), Fiske et al. (2020), Kujala et al. (2020), Jutel and Lupton (2015), Lupton and Jutel (2015), Merz et al. (2018), Powley et al. (2016), Sönnichsen (2019), Morita et al. (2017), Semigran et al. (2015), Thielscher and Antes (2019)
Authority of the technology	4	Meyer et al. (2020), Kujala et al. (2020), Jutel and Lupton (2015), Lupton and Jutel (2015)
Issues of professional power and authority	3	Jutel and Lupton (2015), Lupton and Jutel (2015), Morita et al. (2017)
Irreplaceability of physicians	5	Hill et al. (2020), Wattanapisit et al. (2020), Fiske et al. (2020), Lupton and Jutel (2015), Sönnichsen (2019)
SCA as rival	11	Hill et al. (2020), Razzaki et al. (2018), Wattanapisit et al. (2020), Aboueid et al. (2019), Fiske et al. (2020), Kujala et al. (2020), Jutel and Lupton (2015), Lupton and Jutel (2015), Merz et al. (2018), Thielscher and Antes (2019)
SCA as supplement	11	Miller et al. (2020), Fiske et al. (2020), Kujala et al. (2020), Jutel and Lupton (2015), Lupton and Jutel (2015), Merz et al. (2018), Powley et al. (2016), Sönnichsen (2019), Kuhn et al. (2018), Semigran et al. (2015), Wyatt (2015)
User/patients	35	Hill et al. (2020), Razzaki et al. (2018), Copeland et al. (2018), Millenson et al. (2018), Aboueid et al. (2019), Chambers et al. (2019), Meyer et al. (2020), Miller et al. (2020), Verzantvoort et al. (2018), Fiske et al. (2020), Kujala et al. (2020), Shen et al. (2019), Dunn (2020), Fraser et al. (2018), Jutel and Lupton (2015), Lupton and Jutel (2015), Kramer (2017), Merz et al. (2018), Powley et al. (2016), Ryan and Wilson (2008), Sönnichsen (2019), Morita et al. (2017), Hageman et al. (2015), Kao and Liebovitz (2017), Kuhn et al. 2018, Semigran et al. (2015), Wyatt (2015), Herzog (2019), Lanseng and Andreassen (2007), Nijland et al. (2010), Jimison et al. (2007), Marco-Ruiz et al. (2017), Albrecht et al. (2016), Rowland et al. (2020), Luger et al. (2014)
Acceptance	5	Razzaki et al. (2018), Chambers et al. (2019), Meyer et al. (2020), Verzantvoort et al. (2018), Kuhn et al. (2018)
Adherence and compliance	3	Hill et al. (2020), Chambers et al. (2019), Nijland et al. (2010)
Autonomy	16	Hill et al. (2020), Meyer et al. (2020), Verzantvoort et al. (2018), Fiske et al. (2020), Kujala et al. (2020), Jutel and Lupton (2015), Lupton and Jutel (2015), Merz et al. (2018), Powley et al. (2016), Ryan and Wilson (2008), Sönnichsen (2019), Kao and Liebovitz (2017), Semigran et al. (2015), Nijland et al. (2010), Jimison et al. (2007), Marco-Ruiz et al. (2017)
Awareness of the own body/health	3	Meyer et al. (2020), Fiske et al. (2020), Lupton and Jutel (2015)
Benefits	17	Hill et al. (2020), Razzaki et al. (2018), Copeland et al. (2018), Millenson et al. (2018), Meyer et al. (2020), Miller et al. (2020), Fiske et al. (2020), Fraser et al. (2018), Jutel and Lupton (2015), Lupton and Jutel (2015), Merz et al. (2018), Powley et al. (2016), Ryan and Wilson (2008), Semigran et al. (2015), Wyatt (2015), Nijland et al. (2010), Jimison et al. (2007)
Privacy	10	Hill et al. (2020), Millenson et al. (2018), Aboueid et al. (2019), Fiske et al. (2020), Lupton and Jutel (2015), Kramer (2017), Merz et al. (2018), Kao and Liebovitz (2017), Kuhn et al. (2018), Albrecht et al. (2016)
Concerns regarding privacy and data security	6	Hill et al. (2020), Millenson et al. (2018), Aboueid et al. (2019), Lupton and Jutel (2015), Kramer (2017), Kuhn et al. (2018)
Data sovereignty	2	Kramer (2017), Kuhn et al. (2018)
Deficiencies and risks	7	Fiske et al. (2020), Lupton and Jutel (2015), Kramer (2017), Merz et al. (2018), Kao and Liebovitz (2017), Kuhn et al. (2018), Albrecht et al. (2016)
Risks and potential harm	28	Hill et al. (2020), Razzaki et al. (2018), Copeland et al. (2018), Millenson et al. (2018), Aboueid et al. (2019), Chambers et al. (2019), Meyer et al. (2020), Miller et al. (2020), Verzantvoort et al. (2018), Fiske et al. (2020), Kujala et al. (2020), Shen et al. (2019), Dunn (2020), Fraser et al. (2018), Kramer (2017), Powley et al. (2016), Ryan and Wilson (2008), Sönnichsen (2019), Morita et al. (2017), Hageman et al. (2015), Kao and Liebovitz (2017), Semigran et al. (2015), Wyatt (2015), Lanseng and Andreassen (2007), Marco-Ruiz et al. (2017), Albrecht et al. (2016), Rowland et al. (2020), Luger et al. (2014)
Delayed diagnosis and help	6	Aboueid et al. (2019), Shen et al. (2019), Powley et al. (2016), Ryan and Wilson (2008), Kao and Liebovitz (2017), Albrecht et al. (2016)
Diagnostic (in)accuracy: misdiagnosis	18	Hill et al. (2020), Razzaki et al. (2018), Copeland et al. (2018), Millenson et al. (2018), Aboueid et al. (2019), Chambers et al. (2019), Miller et al. (2020), Fiske et al. (2020), Shen et al. (2019), Dunn (2020), Fraser et al. (2018), Kramer (2017), Powley et al. (2016), Sönnichsen (2019), Morita et al. (2017), Hageman et al. (2015), Semigran et al. (2015), Rowland et al. (2020)
Due to user behaviour and aims	13	Meyer et al. (2020), Miller et al. (2020), Verzantvoort et al. (2018), Fiske et al. (2020), Kujala et al. (2020), Fraser et al. (2018), Kramer (2017), Hageman et al. (2015), Semigran et al. (2015), Wyatt (2015), Lanseng and Andreassen (2007), Albrecht et al. (2016), Luger et al. (2014)
Incorrect use	7	Meyer et al. (2020), Miller et al. (2020), Verzantvoort et al. (2018), Fiske et al. (2020), Fraser et al. (2018), Hageman et al. (2015), Wyatt (2015)
Misconceptions	4	Fiske et al. (2020), Kujala et al. (2020), Kramer (2017), Luger et al. (2014)
Trust in SCA	6	Meyer et al. (2020), Verzantvoort et al. (2018), Kramer (2017), Semigran et al. (2015), Lanseng and Andreassen (2007), Albrecht et al. (2016)
Influences on service use	5	Chambers et al. (2019), Fiske et al. (2020), Ryan and Wilson (2008), Semigran et al. (2015), Marco-Ruiz et al. (2017)
Incorrect triage advice	16	Hill et al. (2020), Razzaki et al. (2018), Copeland et al. (2018), Millenson et al. (2018), Chambers et al. (2019), Verzantvoort et al. (2018), Kujala et al. (2020), Shen et al. (2019), Kramer (2017), Powley et al. (2016), Sönnichsen (2019), Kao and Liebovitz (2017), Semigran et al. (2015), Nijland et al. (2010), Marco-Ruiz et al. (2017), Rowland et al. (2020)
(Patient)safety	9	Aboueid et al. (2019), Chambers et al. (2019), Miller et al. (2020), Verzantvoort et al. (2018), Dunn (2020), Fraser et al. (2018), Semigran et al. (2015), Wyatt (2015), Lanseng and Andreassen (2007)
Psychological harm	5	Miller et al. (2020), Fiske et al. (2020), Ryan and Wilson (2008), Kuhn et al. (2018), Semigran et al. (2015)
**Healthcare system**		
Data protection	6	Millenson et al. (2018), Aboueid et al. (2019), Lupton and Jutel (2015), Kramer (2017), Merz et al. (2018), Kuhn et al. (2018)
Economic value of the data	3	Aboueid et al. (2019), Lupton and Jutel (2015), Merz et al. (2018)
Regulations (privacy policy)	6	Millenson et al. (2018), Aboueid et al. (2019), Lupton and Jutel (2015), Kramer (2017), Merz et al. (2018), Kuhn et al. (2018)
Efficiency gains	19	Hill et al. (2020), Razzaki et al. (2018), Millenson et al. (2018), Aboueid et al. (2019), Chambers et al. (2019), Miller et al. (2020), Fiske et al. (2020), Kujala et al. (2020), Dunn (2020), Fraser et al. (2018), Jutel and Lupton (2015), Lupton and Jutel (2015), Merz et al. (2018), Ryan and Wilson (2008), Kuhn et al. (2018), Semigran et al. (2015), Lanseng and Andreassen (2007), Nijland et al. (2010), Marco-Ruiz et al. (2017)
Increasing efficiency	18	Hill et al. (2020), Razzaki et al. (2018), Millenson et al. (2018), Aboueid et al. (2019), Chambers et al. (2019), Miller et al. (2020), Fiske et al. (2020), Kujala et al. (2020), Dunn (2020), Fraser et al. (2018), Jutel and Lupton (2015), Lupton and Jutel (2015), Merz et al. (2018), Ryan and Wilson (2008), Kuhn et al. (2018), Semigran et al. (2015), Nijland et al. (2010), Marco-Ruiz et al. (2017)
Avoiding unnecessary doctor’s visits	12	Millenson et al. (2018), Aboueid et al. (2019), Chambers et al. (2019), Miller et al. (2020), Kujala et al. (2020), Jutel and Lupton (2015), Lupton and Jutel (2015), Merz et al. (2018), Ryan and Wilson (2008), Semigran et al. (2015), Nijland et al. (2010), Marco-Ruiz et al. (2017)
Increasing the effectiveness of doctor’s visits	2	Kujala et al. (2020), Merz et al. (2018)
Potential cost savings	12	Razzaki et al. (2018), Chambers et al. (2019), Meyer et al. (2020), Miller et al. (2020), Dunn (2020), Lupton and Jutel (2015), Merz et al. (2018), Kuhn et al. (2018), Semigran et al. (2015), Lanseng and Andreassen (2007), Nijland et al. (2010), Marco-Ruiz et al. (2017)
Promoting self-management	3	Merz et al. (2018), Nijland et al. (2010); Marco-Ruiz et al. (2017)
Efficiency losses	17	Hill et al. (2020), Chambers et al. (2019), Meyer et al. (2020), Verzantvoort et al. (2018), Fiske et al. (2020), Kujala et al. (2020), Shen et al. (2019), Dunn (2020), Fraser et al. (2018), Powley et al. (2016), Ryan and Wilson (2008), Sönnichsen (2019), Kao and Liebovitz (2017), Kuhn et al. (2018), Semigran et al. (2015), Marco-Ruiz et al. (2017), Rowland et al. (2020)
Cost increase	3	Fraser et al. (2018), Semigran et al. (2015), Marco-Ruiz et al. (2017)
Increasing health care utilization	13	Meyer et al. (2020), Verzantvoort et al. (2018), Fiske et al. (2020), Kujala et al. (2020), Shen et al. (2019), Dunn (2020), Powley et al. (2016), Ryan and Wilson (2008), Sönnichsen (2019), Kao and Liebovitz (2017), Semigran et al. (2015), Marco-Ruiz et al. (2017), Rowland et al. (2020)
Increase the load on health system/professionals	7	Hill et al. (2020), Chambers et al. (2019), Verzantvoort et al. (2018), Fiske et al. (2020), Kujala et al. (2020), Fraser et al. (2018), Kuhn et al. (2018)
Justice	24	Hill et al. (2020), Razzaki et al. (2018), Copeland et al. (2018), Millenson et al. (2018), Aboueid et al. (2019, Chambers et al. (2019), Meyer et al. (2020), Miller et al. (2020), Fiske et al. (2020), Kujala et al. (2020), Jutel and Lupton (2015), Lupton and Jutel (2015), Kramer (2017), Ryan and Wilson (2008), Morita et al. (2017), Hageman et al. (2015), Kao and Liebovitz (2017), Kuhn et al. (2018), Wyatt (2015), Lanseng and Andreassen (2007), Nijland et al. (2010), Marco-Ruiz et al. (2017), Luger et al. (2014), Thielscher and Antes (2019)
Improved access	15	Razzaki et al. (2018), Copeland et al. (2018), Aboueid et al. (2019), Meyer et al. (2020), Miller et al. (2020), Fiske et al. (2020), Jutel and Lupton (2015), Lupton and Jutel (2015), Kramer (2017), Morita et al. (2017), Kao and Liebovitz (2017), Kuhn et al. (2018), Wyatt (2015), Lanseng and Andreassen (2007), Thielscher and Antes (2019)
Advantageous for structurally weak regions	8	Copeland et al. (2018), Aboueid et al. (2019), Fiske et al. (2020), Lupton and Jutel (2015), Morita et al. (2017), Kao and Liebovitz (2017), Kuhn et al. (2018), Thielscher and Antes (2019)
Advantageous for specific groups	2	Meyer et al. (2020), Wyatt (2015)
Broader access to healthcare in general	10	Razzaki et al. (2018), Aboueid et al. (2019), Meyer et al. (2020), Miller et al. (2020), Fiske et al. (2020), Jutel and Lupton (2015), Lupton and Jutel (2015), Kramer (2017), Kuhn et al. (2018), Lanseng and Andreassen (2007)
Influencing factors	15	Hill et al. (2020), Millenson et al. (2018), Aboueid et al. (2019), Chambers et al. (2019), Meyer et al. (2020), Miller et al. (2020), Kujala et al. (2020), Kramer (2017), Hageman et al. (2015), Kuhn et al. (2018), Wyatt (2015), Lanseng and Andreassen (2007), Nijland et al. (2010), Marco-Ruiz et al. (2017), Luger et al. (2014)
Access to technology	2	Kujala et al. (2020), Wyatt (2015)
Digital literacy	6	Hill et al. (2020), Miller et al. (2020), Kujala et al. (2020), Kuhn et al. (2018), Lanseng and Andreassen (2007), Luger et al. (2014)
Health literacy	4	Hill et al. (2020), Kramer (2017), Marco-Ruiz et al. (2017), Luger et al. (2014)
Regarding decisions to use	8	Aboueid et al. (2019), Chambers et al. (2019), Meyer et al. (2020), Miller et al. (2020), Kujala et al. (2020), Kramer (2017), Hageman et al. (2015), Nijland et al. (2010)
Regarding user behaviour	5	Millenson et al. (2018), Aboueid et al. (2019), Chambers et al. (2019), Meyer et al. (2020), Luger et al. (2014)
Usability	5	Miller et al. (2020), Kujala et al. (2020), Kramer (2017), Lanseng and Andreassen (2007), Luger et al. (2014)
Potential for discrimination	3	Chambers et al. (2019), Meyer et al. (2020), Ryan and Wilson (2008)
Structural changes	3	Verzantvoort et al. (2018), Fiske et al. (2020), Lupton and Jutel (2015)
Boundaries of the clinical setting	1	Fiske et al. (2020)
Medicalisation/healthism	2	Verzantvoort et al. (2018), Lupton and Jutel (2015)

^a^Frequency of the references in which the (sub-)category was coded at least once (n = 39)

### Technology

We subsumed text passages that generally concern the development process of SCA, its context and outcomes under the main category *Technology*. This category comprises the seven subcategories: *Commercial Aspects, Expertise of Producers/Developers, Lack of Evidence, Lack of Regulation, (In)Transparency, Quality of the Apps* and *User Participation in Development.*

In the subcategory *Commercial Aspects* (Ryan and Wilson 2008; Jutel and Lupton 2015; Lupton and Jutel 2015; Powley et al. 2016; Kramer 2017; Merz et al. 2018; Fraser et al. 2018; Sönnichsen 2019; Fiske et al. 2020; Dunn 2020), we addressed the conflict between ‘[…] the business interests of device manufactures [and] good patient outcomes’ (Fiske et al. 2020). Issues discussed in this context are, for instance, commercial sponsoring of the app developers (Lupton and Jutel 2015), ‘commercial interests’ (Lupton and Jutel 2015) and ‘promotional purposes’ (Lupton and Jutel 2015) of the app provider, financial collaborations between the app provider and other companies, and SCA as a part of marketing efforts (Lupton and Jutel 2015; Ryan and Wilson 2008). The sale of user data to third parties for commercial use (Lupton and Jutel 2015) and the independence of accompanying research (Fraser et al. 2018; Merz et al. 2018; Sönnichsen 2019) were also examined. For instance, some apps ‘[…] provide contacts to actual physicians should users decide that their symptoms warrant further investigation. In this app, a list of doctors is provided for real-time contact, paid for by the minute. As such, the app acts as a conduit for promoting doctors’ services in ways that are not readily apparent from first appraisal of the app’s content and purpose’ (Lupton and Jutel 2015). Another example is that apps may lead to ‘[…] advice to buy a health insurance policy or use an online pharmacy that is partnered with the company, and that [the app provider] may receive financial compensation for such referrals and affiliate programs’ (Lupton and Jutel 2015). The lack of information regarding these commercial interests and associated conflicts of interest are criticized in the literature (Jutel and Lupton 2015; Lupton and Jutel 2015). ‘Identifying and mitigating the effects of financial conflicts of interest related to symptom checkers is [seen by some authors as] an unresolved challenge’ (Dunn 2020) and ‘[t]he commercial interests underpinning self-diagnosis [apps are seen as requiring] further investigation’ (Lupton and Jutel 2015).

These aspects are closely linked to the debate in the subcategory *Expertise of Producers/Developers* (Jutel and Lupton 2015; Lupton and Jutel 2015; Morita et al. 2017; Verzantvoort et al. 2018; Hill et al. 2020), in which we collated citations regarding the common problem that app developers and producers are often not ‘medical experts’ (Lupton and Jutel 2015) or ‘reputable organisations’ (Hill et al. 2020), but entrepreneurs with interests outside medicine. The lack of appropriate (medical) credentials is criticized (Lupton and Jutel 2015; Hill et al. 2020) insofar as many apps made no statements about their authorship or simply refer to a ‘doctor’ or a medical team in the app description without providing more details or evidence (Lupton and Jutel 2015). ‘For example, both the app description and the developer’s website for [the symptom checker] make mention of the app content being contributed by the prestigious Harvard Medical School, but no further details are supplied to support this claim’ (Lupton and Jutel 2015). Medical and scientific authority is used in these examples to render the apps a serious contender. This involves the risk that the information contained in the apps, and consequently the results of the apps, are not backed by medicine and science to the degree they appear to be. App users could easily be deceived and false expectations could be raised. Against this background, some authors call for an active involvement of medical professionals in the development process of SCA to establish them on a professional basis and also improve them (Morita et al. 2017). Whether such measures would be helpful against misinformation, or whether more regulation or specific licensing procedures would be necessary, remains to be discussed.

In the subcategory *Lack of Evidence* (Ryan and Wilson 2008; Hageman et al. 2015; Semigran et al. 2015; Wyatt 2015; Kao and Liebovitz 2017; Kramer 2017; Millenson et al. 2018; Fraser et al. 2018; Kuhn et al. 2018; Merz et al. 2018; Chambers et al. 2019b; Sönnichsen 2019; Aboueid et al. 2019; Kujala et al. 2020; Dunn 2020; Iacobucci 2020), it is strongly emphasized by several authors that the ‘accuracy of symptom checkers is still under question’ (Aboueid et al. 2019) and that scientific evidence regarding safety and efficacy is limited (Kao and Liebovitz 2017; Kramer 2017; Kuhn et al. 2018; Millenson et al. 2018; Sönnichsen 2019; Kujala et al. 2020). Some publications refer to comparisons of the apps’ performance with that of health professionals (Hageman et al. 2015; Chambers et al. 2019a; Dunn 2020), but with the caveat that these results are limited and should be interpreted with caution (Millenson et al. 2018; Chambers et al. 2019a). Proving the quality of an app due to a lack of methods is highlighted as a problem (Kramer 2017). In addition, it is questioned whether existing methods are adequate for the assessment of the apps or whether new methods for evaluation need to be developed (Merz et al. 2018). In this regard, several authors demand further research and more robust evaluations regarding safety, efficacy, and effectiveness (Wyatt 2015; Millenson et al. 2018; Fraser et al. 2018; Iacobucci 2020).

In the publications, this debate is often followed by discussions on regulatory gaps around these technologies, which we subsumed in the subcategory *Lack of Regulation* (Fraser et al. 2018; Kao and Liebovitz 2017; Fiske et al. 2020; Dunn 2020; Iacobucci 2020). Here, the problem is addressed that SCA ‘are typically not subject to the same level of regulation’ (Dunn 2020) as other software (for clinical diagnosis) or medical devices, because many SCA circumvent this regulation by maintaining that they do not provide diagnoses and medical advice (Dunn 2020; Iacobucci 2020). Some authors state the concern that regulatory gaps ‘could compromise users’ health and safety’ (Kao and Liebovitz 2017) and create ethical problems, for example, for physicians who want to care for patients who use unregulated devices (Fiske et al. 2020). According to these authors, approval by governing bodies, ongoing monitoring, regulatory frameworks, and certification procedures are definitely required (Kao and Liebovitz 2017; Fraser et al. 2018; Dunn 2020).

This debate is also closely linked to the text fragments that we collated in the subcategory *(In)Transparency* (Lanseng and Andreassen 2007; Lupton and Jutel 2015; Kramer 2017; Aboueid et al. 2019; Herzog 2019; Sönnichsen 2019; Hill et al. 2020). The accusation of intransparency refers to the apps’ descriptions, their presentations on the websites, terms of use, authorship, and interests that may lie behind these apps (Hill et al. 2020; Lupton and Jutel 2015). Some authors criticize that the relevant information is often not apparent at first sight for the user, difficult to understand (Kramer 2017), or in some cases not provided at all (Lupton and Jutel 2015). The responsibility of the app developer and the trust of the user are normative concepts referred to in this context (Kramer 2017).

A further concern debated in the subcategory *Quality of the Apps* (Ryan and Wilson 2008; Jutel and Lupton 2015; Lupton and Jutel 2015; Millenson et al. 2018; Fraser et al. 2018; Fiske et al. 2020) is the varying quality of the apps, the accuracy and reliability of the information used in the apps (Lupton and Jutel 2015), and the validity of the outcome (Lupton and Jutel 2015). In this context, some authors claim that the apps should be better designed, tested, and evidence-based (Ryan and Wilson 2008) and they propose different research designs for this, such as pre-deployment testing, user-interaction studies, or field trials (Fraser et al. 2018). The authors criticize furthermore that there are no ‘[…] accepted vetting processes enabling clinicians or patients to distinguish between reliable apps and “digital snake oil”’ (Millenson et al. 2018).

Some authors consider patient engagement necessary in the development of the apps. User participation in the development and research of SCA is addressed in the subcategory *User Participation in Development* (Lanseng and Andreassen 2007; Kao and Liebovitz 2017; Aboueid et al. 2019; Kujala et al. 2020). With this in mind, authors claim as important: Firstly, to ensure a diverse sample of users in the test phases of the apps (Aboueid et al. 2019; Lanseng and Andreassen 2007) and, secondly, to convince the public that might be sceptical about this technology (Aboueid et al. 2019). A further argument raised in this context, is to study the patients’ experience to identify their needs and barriers of use (Kujala et al. 2020).

### Individual Level

The second main category *Individual Level* consists of three subcategories and refers to aspects regarding healthcare professionals, end-users (respective patients), and their relationships to each other.

In the subcategory *Healthcare Professionals,* we included text passages on the role, expertise, and responsibility of healthcare professionals, in particular, regarding diagnostic processes (Lupton and Jutel 2015; Semigran et al. 2015; Kramer 2017; Kuhn et al. 2018; Merz et al. 2018; Razzaki et al. 2018; Aboueid et al. 2019; Sönnichsen 2019; Hill et al. 2020; Wattanapisit et al. 2020; Fiske et al. 2020; Kujala et al. 2020; Dunn 2020). The underlying questions of the discussions are specifically whether SCA require changes within the healthcare professions, could replace medical professionals, or even lead to a devaluation of the professions. With slogans such as ‘Dr. Google v Dr. Human’ (Fiske et al. 2020) the authors debate which aspects constitute a diagnosis, which skills are needed for diagnostic processes (e.g., experience, empathy, and time), the adequate place of the apps in the diagnostic process, and whether the apps could replace the physician (Lupton and Jutel 2015; Merz et al. 2018; Hill et al. 2020). On the one hand, the performance of physicians is seen as ‘the gold-standard’ (Razzaki et al. 2018). The authors describe certain skills of physicians, such as understanding and empathy as unique and, hence, irreducible (Sönnichsen 2019; Dunn 2020). Moreover, the authors underline that diagnoses require medical training and professional expertise (Fiske et al. 2020). On the other hand, several authors see the potential of these apps to change the social dynamic around diagnostic processes (Lupton and Jutel 2015) and thereby the role and self-concept of healthcare professionals (Kuhn et al. 2018; Merz et al. 2018). The authors present digital expertise as a new but essential skill, which is needed by physicians evaluating the apps in order to help patients with the use of these new technologies (Kuhn et al. 2018; Kujala et al. 2020). In this regard, more awareness, education, and support for healthcare professionals are demanded by some authors (Semigran et al. 2015; Kuhn et al. 2018; Merz et al. 2018; Kujala et al. 2020).

The subcategory *User/Patients* comprises all text passages that refer to aspects regarding the users of SCA, ranging from their acceptance of the technology, to possible shifts in the awareness of their own health, to the benefits and risks for the users employing the app (Jimison et al. 2007; Lanseng and Andreassen 2007; Ryan and Wilson 2008; Nijland et al. 2010; Luger et al. 2014; Hageman et al. 2015; Jutel and Lupton 2015; Lupton and Jutel 2015; Semigran et al. 2015; Wyatt 2015; Albrecht et al. 2016; Powley et al. 2016; Kao and Liebovitz 2017; Kramer 2017; Marco-Ruiz et al. 2017; Morita et al. 2017; Kuhn et al. 2018; Merz et al. 2018; Razzaki et al. 2018; Copeland et al. 2018; Fraser et al. 2018; Millenson et al. 2018; Verzantvoort et al. 2018; Aboueid et al. 2019; Chambers et al. 2019b; Shen et al. 2019; Sönnichsen 2019; Hill et al. 2020; Meyer et al. 2020; Miller et al. 2020; Fiske et al. 2020; Kujala et al. 2020; Dunn 2020; Rowland et al. 2020).

We noted many contradictory statements in the subcategory User/Patients. The limited evidence regarding the users’ perspectives on the apps, their satisfaction (Chambers et al. 2019b) and compliance with the apps’ advice (Nijland et al. 2010; Chambers et al. 2019b) is addressed. Concerning the users’ autonomy, the authors of the publications debate whether digital applications could contribute to the greater empowerment and independence of users and patients (Fiske et al. 2020). Potential is seen in the improvement of users’ interest, engagement, and understanding (Fiske et al. 2020). The apps might help to assess medical information and the own symptoms, assist in decision-making and seeking help (Lupton and Jutel 2015), support self-care (Marco-Ruiz et al. 2017; Kujala et al. 2020), and allay anxieties (Lupton and Jutel 2015). The users might be able to educate themselves and improve their awareness of their own health (Hill et al. 2020).

By contrast, other authors state that the apps do not increase the autonomy of the users, but instead their feelings of uncertainty (Sönnichsen 2019). Consequently, these authors make the requirement for further evaluations of SCA and a more ‘in-depth evaluation of patients’ needs for autonomy and their readiness to make decisions about their health care’ (Nijland et al. 2010). Further evaluations should serve to assess whether the apps help the users to make better decisions about their health. In addition, the evaluations should show how the use of these apps affects the well-being of the users (Semigran et al. 2015).

The use of terms such as ‘autonomy’ and ‘empowerment’ in the advertising of the apps is also addressed by some authors. The app user is often characterised by the app provider (e.g. in the context of the app advertising) as the ‘digitally engaged patient’ (Lupton and Jutel 2015), referring to ‘the ideal of the empowered patient’ (Lupton and Jutel 2015). This goes so far that the user is described as a medical expert or their ‘own physician’ (Merz et al. 2018). Some authors find it problematic that ‘patient engagement’ and ‘empowerment’ is also a ‘hallmark of digital health rhetoric’ by the app providers (Lupton and Jutel 2015).

Besides issues of autonomy, general benefits for the app users are discussed in the publications: immediate, mobile, more convenient, and more targeted information (Ryan and Wilson 2008; Copeland et al. 2018; Millenson et al. 2018); earlier disease and risk detection (Nijland et al. 2010); reduction of diagnostic errors (Miller et al. 2020); direction to the appropriate care setting (Semigran et al. 2015); timely care and, hence, better outcomes, particularly for emergencies and urgent health conditions (Hill et al. 2020). In general, the fulfilment of patients’ needs (Miller et al. 2020) and saving users’ time and money (Lupton and Jutel 2015; Miller et al. 2020) are listed as benefits of the app use.

At the same time, many risks are addressed in the publications. Especially, concerns about the safety, applicability, and the correct use of the apps are raised (Verzantvoort et al. 2018). Because of significant variation in the assessment of symptoms in terms of clinical accuracy (Semigran et al. 2015; Powley et al. 2016; Razzaki et al. 2018; Shen et al. 2019), the authors are concerned about harm to the patients. Negative consequences for patients could include, for example, delayed verified medical diagnoses (Albrecht et al. 2016; Kao and Liebovitz 2017; Aboueid et al. 2019), deferred seeking of help (Powley et al. 2016; Shen et al. 2019), diagnostic errors (Millenson et al. 2018), and following incorrect treatment advice (Aboueid et al. 2019).

The results of this review indicate a mixed picture about triage advice (Semigran et al. 2015; Razzaki et al. 2018; Shen et al. 2019). Several studies indicate that SCA are rather risk-averse (Sönnichsen 2019; Chambers et al. 2019b) and insofar provide advice that is not clinically appropriate (Chambers et al. 2019b), e.g. unnecessary consultations of a physician. Again, we perceive some ambivalence in the literature: The authors highlight that risk-averse apps can burden the healthcare systems further, but the opposite (less careful apps) can be dangerous for the users (Kramer 2017; Marco-Ruiz et al. 2017; Hill et al. 2020); for instance, if the app suggested staying at home when (urgent) treatment was required. At the same time, it is stated that there is no evidence whether or not SCA are detrimental to patient safety (Chambers et al. 2019b).

Some authors also emphasize the psychological burden through app use: SCA could lead to uncertainties and further anxieties due to misdiagnosis and mistriage (Kuhn et al. 2018; Ryan and Wilson 2008). A psychological consequence could be ‘cyberchondria’ (Fiske et al. 2020), which is described as the reinforcement of health anxieties due to extensively searching for health-related information (Ryan and Wilson 2008; Semigran et al. 2015).

Further harm to the users can be caused by the issue that ‘patients may not be able to appropriately interpret and contextualize data’ (Fiske et al. 2020), may ignore correct advice by the app (Verzantvoort et al. 2018), or neglect other treatments because of the app use (Kramer 2017). Aspects of responsibility for one’s own health were not found in the texts. Other concerns expressed in the publications relate to data security and user privacy (Lupton and Jutel 2015; Kramer 2017; Millenson et al. 2018), in particular, highlighting the sensitivity of (health) data (Lupton and Jutel 2015; Kuhn et al. 2018).

In the subcategory *User/Patient-Physician Relationship*, we included all aspects regarding the relationship between the user and medical professionals, especially characteristics of this relationship, such as collaboration (Fiske et al. 2020), in-person contact, conversation (Miller et al. 2020; Fiske et al. 2020), and clinical rapport (Wyatt 2015). Several authors mention potential effects on these relationships due to app use (Jutel and Lupton 2015; Lupton and Jutel 2015; Aboueid et al. 2019; Hill et al. 2020; Fiske et al. 2020), for example, fundamental changes to treatment processes (Kuhn et al. 2018) or users’ concerns about their physicians’ reactions (Meyer et al. 2020). Predominantly, issues of professional power and authority are discussed (Jutel and Lupton 2015; Meyer et al. 2020) as SCA are often represented by the app producers as having algorithmic and medical authority (Meyer et al. 2020). In this context, the authors discuss whether SCA could replace medical professionals in the long run or whether the professionals’ expertise is irreplaceable and SCA can only have a supplementary function (Jutel and Lupton 2015; Morita et al. 2017; Merz et al. 2018; Fiske et al. 2020; Hill et al. 2020; Meyer et al. 2020; Wattanapisit et al. 2020). Some publications take up the concern of healthcare professionals that SCA constitute a threat to their professional autonomy and role because SCA could potentially replace their expertise and reduce their control (Fiske et al. 2020; Kujala et al. 2020).

### Healthcare system

In the third main category *Healthcare System,* we gathered aspects relevant at the institutional level of the healthcare system. We constructed five subcategories: *Data Protection, Efficiency Gains, Efficiency Losses, Justice,* and *Structural Changes*.

In the subcategory *Data Protection,* we addressed problems that arise from the economic value of the data collected by SCA (Lupton and Jutel 2015; Aboueid et al. 2019; Merz et al. 2018). ‘Ethical concerns surrounding the digital economy’ (Aboueid et al. 2019) and the ‘monetisation of digital data’ (Lupton and Jutel 2015) are the main concerns in this subcategory. Many app developers and providers are profit-oriented companies that see diagnostic apps as a lucrative new market segment (Merz et al. 2018). It is criticized, that these developers and providers sell the users’ data to third parties for profit (Lupton and Jutel 2015). With this in mind, the scope of the users’ informed consent and the possibilities of opting out should be carefully examined (Merz et al. 2018). In addition, more transparency for users, for example with regard to data management, is demanded (Aboueid et al. 2019). The current deficiencies of the apps regarding data protection and the current lack of sufficient regulations is one of the main points of criticism (Lupton and Jutel 2015; Kramer 2017; Kuhn et al. 2018; Merz et al. 2018; Aboueid et al. 2019). Although regulations are emphatically demanded regarding data protection and security, regulations should also not hinder innovation in this area. The balance between enabling technological innovations and preventing them through overregulation is a controversial issue in the publications (Millenson et al. 2018; Aboueid et al. 2019).

Text passages in the subcategory *Efficiency Gains* refer to improvements for the healthcare systems by SCA use in general (Fraser et al. 2018; Razzaki et al. 2018). A potential improvement discussed in the literature is expediting emergency care (Kuhn et al. 2018; Chambers et al. 2019b; Hill et al. 2020; Miller et al. 2020). A further main advantage of the app use is seen in the possibility that the use of SCA enables self-care and self-management (Nijland et al. 2010; Marco-Ruiz et al. 2017; Merz et al. 2018) and, hence, avoiding unnecessary visits to physicians (Ryan and Wilson 2008; Nijland et al. 2010; Lupton and Jutel 2015; Semigran et al. 2015; Marco-Ruiz et al. 2017; Kuhn et al. 2018; Merz et al. 2018; Aboueid et al. 2019; Chambers et al. 2019b; Kujala et al. 2020). Further improvements are seen in the increase of the effectiveness of triage (Kuhn et al. 2018; Hill et al. 2020), which means that patients would be assigned to the appropriate treatment more quickly, and thus saving costs (Lanseng and Andreassen 2007; Nijland et al. 2010; Lupton and Jutel 2015; Marco-Ruiz et al. 2017; Merz et al. 2018; Razzaki et al. 2018; Aboueid et al. 2019; Chambers et al. 2019b; Dunn 2020; Meyer et al. 2020; Miller et al. 2020; Kuhn et al. 2018).

Nonetheless, we also found aspects that could lead to a greater burden on healthcare systems in the subcategory *Efficiency Losses:* reaching from unnecessary consultations due to risk-averse SCA (Chambers et al. 2019b; Fiske et al. 2020; Kujala et al. 2020), followed by an increased workload for medical professionals, to rising costs for society (Semigran et al. 2015; Powley et al. 2016; Kao and Liebovitz 2017; Marco-Ruiz et al. 2017; Verzantvoort et al. 2018; Sönnichsen 2019; Dunn 2020; Hill et al. 2020; Kujala et al. 2020; Meyer et al. 2020; Rowland et al. 2020). It is criticized by the authors that SCA could encourage users to seek professional care even when self-care would be suitable or advise patients to attend hospital rather than consult a primary-care physician. The SCA were seen as additional work for medical professionals as they create one more channel to handle. The authors note additionally that the rise in demand for services that were not medically indicated could unduly burden healthcare systems (Fiske et al. 2020).

Following on the heels of these two contrasting categories, the subcategory *Justice* comprises elements of the discourse that deal with issues of justice in relation to healthcare. Many text passages address the potential of SCA to improve the access to healthcare, but often without further specification and few cross-references to health-service research indicators (Lupton and Jutel 2015; Kramer 2017; Razzaki et al. 2018; Aboueid et al. 2019; Meyer et al. 2020; Miller et al. 2020). Some authors acknowledge a better provision of healthcare for structurally weak regions or resource-limited settings, such as in rural areas or developing countries (Lupton and Jutel 2015; Morita et al. 2017; Kao and Liebovitz 2017; Copeland et al. 2018; Kuhn et al. 2018; Aboueid et al. 2019; Thielscher and Antes 2019; Fiske et al. 2020). A better provision of healthcare might also be engendered for certain populations such as underrepresented groups or patients with anxiety (Wyatt 2015; Meyer et al. 2020).

By contrast, some authors are concerned that the use of SCA could lead to decreased access and contains the potential for discrimination. In this context, the authors discuss socio-demographic factors, such as age (Luger et al. 2014; Miller et al. 2020), gender, or education (Hageman et al. 2015; Aboueid et al. 2019; Chambers et al. 2019b) as potentially influential factors. Especially, health and digital literacy are focussed on (Ryan and Wilson 2008; Kramer 2017; Marco-Ruiz et al. 2017; Hill et al. 2020; Miller et al. 2020). Access to the technology and the usability of the apps (Lanseng and Andreassen 2007; Kujala et al. 2020; Meyer et al. 2020) are mentioned as further potentially influential aspects*.* A discriminatory situation may arise when pathways of care prioritise digital requests over telephone requests (Chambers et al. 2019b) since mobile phone use for health information may vary across age, gender, and ethnicity (Luger et al. 2014; Meyer et al. 2020). Some publications suggest that younger and more highly-educated individuals are more likely to use SCA while older and less educated persons are more likely to prefer telephone or face-to-face contact (Luger et al. 2014; Chambers et al. 2019b). Some authors express the concern, therefore, that SCA use ‘could lead to increased inequity in healthcare provision by further empowering well-educated affluent groups at the expense of people from less affluent groups’ (Ryan and Wilson 2008). In this context, the keywords ‘digital divide’ (Kuhn et al. 2018) and ‘health (in)equity’ (Ryan and Wilson 2008; Chambers et al. 2019b; Kujala et al. 2020) are alluded to but without further definition or explanation.

In addition, we collected possible changes at the institutional and structural level of healthcare systems in the subcategory *Structural Changes*. The implications of SCA use might result in phenomena such as ‘medicalisation’ (Verzantvoort et al. 2018) or ‘healthism’ (Lupton and Jutel 2015), but these concepts are not explained further in the respective publications. However, the publications describe one main shift resulting from the use of SCA, namely the possibility to assess bodily phenomena in a manner that was previously impossible outside of clinical settings (Fiske et al. 2020). Consequently, the boundaries of the clinical setting are shifted according to the authors. ‘These practices mark the changing boundaries of what is considered “clinical”, as they no longer require patients to physically enter a specific professional or technological space: The clinic is no longer something that they go to, but something that comes to them’ (Fiske et al. 2020). Although this potential shift is addressed by some authors, it is not clear whether this shift is assessed negatively or positively by them.

## Discussion

This review aimed to identify the ethical, legal, and social implications of consumer-targeted SCA in the respective scientific literature and to provide an overview on the spectrum of corresponding issues discussed therein.

The results of the review show that SCA may have implications for specific ethical, legal, and social aspects. As we argued in the introduction to this article, apps on mobile devices have typical features that require a specific analysis. However, these characteristics are rarely discussed in any specific detail in the literature. SCA are not often examined on their own, but in the broader context of other mobile health technologies or apps. Many publications do not distinguish which type of app is being studied. This runs the risk of losing sight of the particular ethical, legal, and social challenges associated with SCA. However, the relevance of discussing SCA and their implications separately is also demonstrated by the amount of ELSA, research gaps, and controversial discussions we could identify in this review.

While many of the aspects such as the regulation of SCA, privacy, or the respective responsibilities of stakeholders were mentioned with reference to legal debates and areas in which legal issues must still be explored or clarified, no specific legal literature regarding SCA could be identified in the screened publications. Although there are aspects which are part of legal debates such as privacy, these aspects were framed as ethical rather than as legal issues in the publications and were not discussed from a specific jurisprudential perspective (meaning that these aspects were not discussed by lawyers or in strictly legal terms). In short: legal aspects were found, but no legal argumentation. In the wider context of mHealth, however, there is an ongoing jurisprudential debate on the legal aspects of health apps. In addition to inadequate regulation and insufficiently clarified data-protection issues, liability and malpractice are also addressed. Despite these discussions, however, no pertinent legal literature considering SCA specifically could be identified in the current review. Besides the research methodology (search strings and databases), various reasons may explain this gap in the literature. For one, SCA do not yet appear to have been identified as a distinct area within mHealth. For another, no controversial legal case due to the use of SCA has claimed the attention of scholars of law, as far as we know. It may be speculated that this is either due to the underdeveloped stage and obvious faults of SCA or due to users still prioritising a visit to their general practitioner by habit. However, questions of approval as a medical device were found in the screened texts. In the analysed publications, the regulatory gaps related to SCA were frequently stated, for example, the lower level of regulation of SCA compared to other (medical) software.

This review highlights both the regulatory gaps in practice and the missing jurisprudential literature specific to SCA. Both ought to be addressed in further research. In the collaborative CHECK.APP project, we will conduct interviews with legal experts to analyse the gaps in the German debate and practice. Legally relevant issues such as privacy, data protection, and patient autonomy will have to be transferred to a legal debate, considering the respective legal and healthcare systems of the individual countries. Solutions to the regulatory gaps that ought to be considered include licensing and certification procedures, ongoing monitoring, and a legal framework.

The results of the review also indicate that there is little consensus on the positive and negative effects of SCA, but rather that widely differing positions exist which lead to different evaluations of this new technology. For example, a variety of reasons is given in the literature analysed in favour of the implementation and use of SCA, such as the benefits for the end-users and the healthcare systems. Concomitantly, many arguments are made against the use of SCA, such as additional burdens for the end-users and the healthcare systems. These seemingly contradictory views might be due to the absence of comparable empirical data as well as different healthcare systems and regulatory frameworks under which SCA exert their beneficial or more detrimental effects. However, the differing views on the impact of SCA may not be incompatible after all, as the potentially increased burdens may be the price to be paid for the benefits that SCA may ultimately bring. For example, additional burdens on end-users may be acceptable in light of the benefits that SCA would bring to physicians or healthcare systems.

In any case, such a strong divergence shows that further research is imperative to obtain a comprehensive overview of the complex and multi-facetted issues of SCA. Further analyses are essential to ascertain the decisive factors in the tableau of arguments that led certain authors in this discourse to consider SCA use as acceptable and beneficial under certain conditions, while others concluded that the disadvantages and risks prevail. There may be a decided research bias because disciplines rarely engage in mutual exchange. Future research ought to examine the different premises and perspectives underlying the respective arguments in detail and how these arguments are weighted in the different evaluations of this new technology. In two subprojects of the CHECK.APP project, we will conduct interviews with primary healthcare providers as well as experts of the German healthcare system to map out the various positions in the German context (Wetzel et al. 2022).

This review also establishes that the discussions on the ethical, legal, and social implications of SCA are often poorly supported by empirical data. Most of the reflections on SCA are based on a hypothetical widespread use of the apps. Even though the rapid developments in the field of mHealth suggest an increasing use, SCA are still at an early stage of commercialisation and their use is not pervasive yet. Considering that SCA are an emerging technology, much of the literature discussed has a prognostic character and anticipates potential developments in the use of SCA. As the articles often base their arguments on a hypothetical broad deployment of SCA, these arguments should be taken with caution and further research should test the plausibility of their predictions. Without empirical data, a well-informed discussion of the ethical, legal, and social aspects is possible but remains hypothetical.

When empirical studies are referred to in the screened publications, they are often the same few empirical studies [e.g., Semigran et al. 2015]. This bears the risk of bias: the results of the few studies may be amplified. A corrective measure could be the search for studies in further disciplines, unpublished empirical data, or grey literature. Some reviews about the performance and evidence of digital and online applications for diagnosis already exist (Millenson et al. 2018; Wattanapisit et al. 2020) but, in general, evidence is limited and methodological challenges arise regarding the standards of comparison, reproducibility, and interpretation. Further empirical research on the use and distribution of the apps, their possible implications for different levels of healthcare provision, and the perspectives of end-users, practitioners, and developers are thus needed for a well-informed debate. Our collaborative CHECK.APP project will include several sub-projects that aim to generate evidence for different perspectives in this context, for example, the perspectives of SCA users, general practitioners, and experts (Wetzel et al. 2022).

## Limitations

Although this review employed various databases, only literature was used that was freely available or accessible to the research team through their institutions. Further databases, for instance, with a focus on psychological aspects or engineering issues, might have led to the identification of further pertinent literature. Especially literature about the legal challenges might still remain “concealed” in other databases, so that the discussion—at least in Germany—might be mapped differently if this literature was identified and cited. Although we searched in international databases such as Web of Science or PubMed, our research was conducted—at least in part—from a European perspective. For example, the legal databases Juris and BeckOnline focus on European and German research. Further research could extend this mostly European perspective to a global level, for instance, and also compare the ‘landscapes’ of different healthcare systems and jurisdictions.

Although many (smartphone-based) SCA are freely available in online application stores (e.g., Apple’s App Store), most of them have not been discussed in the scientific literature. Other data sources outside the scientific setting such as blogs or app descriptions could therefore provide further information. In addition to the chosen databases, the selected search strings also lead to limitations as well as the exclusion of literature not written in English or German. Furthermore, the literature mirrors the heterogeneity of the healthcare systems in which the research was conducted. This puts limits especially on the legal aspects and the applicability of the normative debate to a concrete healthcare context. We hope that the CHECK.APP project’s focus on one setting, namely the German healthcare system, proves to be an asset.

The reviewers’ understanding of the ethical, legal, and social aspects is what this review hinges on. A very broad understanding, based on the reviewers’ training and research experience, was used in order not to limit the variety of aspects identified. In addition, deciding whether a publication or text fragment matches the framework in the qualitative data synthesis is essentially a subjective process. However, these decisions were rendered intersubjective and transparent by bringing together researchers from different disciplines, discussing relevant decisions within the interdisciplinary research team, and resolving disagreements through discourse (Smaling 1992).

## Conclusions

The results of this review show that there are very different, sometimes even controversial, ethical, legal, and social challenges regarding the responsible usage of SCA. Due to the variety of the ethical and social aspects in particular, paired with a missing legal debate and a lack of empirical data, it is difficult to derive general recommendations regarding the usage of SCA in an everyday health context from this review. Instead, the review indicates further discussion and careful examination of the validity of potential recommendations and abstaining from general recommendations before further research has been conducted. However, the review shows which issues such recommendations need to address and on what kind of empirical basis they need to be founded. Furthermore, the ethical, legal, and social aspects presented in this review may be considered when healthcare professionals care for patients who use SCA or are asked for help and recommendations in these matters.

In addition, the overview presented in this review can provide the basis for further research. Empirical studies on the views about these technologies by different stakeholders, such as users, patients, and healthcare professionals would supplement this review. Our collaborative CHECK.APP project aims to provide a comprehensive analysis of the German context with regard to the perspectives of patients, general practitioners, and experts of the healthcare system. Future research may lead to more concrete implications for practice, such as what kind of consequences these technologies entail for the relationship between patients and medical practitioners. Furthermore, future research can address the missing legal discussion and fill in the regulatory gaps and taking the respective legal systems and the specificities of the healthcare systems of the individual countries into account.

## Supplementary Information

Below is the link to the electronic supplementary material.

Supplementary file1 (DOCX 107 KB)

Supplementary file2 (DOCX 24 KB)

## Data Availability

All data analysed during this review are included in this published article and its supplementary information files. (Further data that support the findings of this study are available from the corresponding author on reasonable request.)

## Abbreviations

ELSA: Ethical, legal, and social aspects
SCA: Symptom Checker Applications

## Footnotes

1 In the context of SCA, the term "triage" refers to the referral of users to a suitable department for the most appropriate treatment or for further investigation within a reasonable timeframe. The appropriate treatment or investigation depends on the nature and urgency of the symptoms or their underlying cause.
