# Supplementary material for: Ethical, legal, and social aspects of symptom checker applications: a scoping review
Source: Med Health Care Philos. 2022 Oct 1;25(4):737–55. doi: 10.1007/s11019-022-10114-y (PMC9613552; doi:10.1007/s11019-022-10114-y)
Supplement: Supplementary file 2 — Supplementary file2 (DOCX 24 KB) [file 11019_2022_10114_MOESM2_ESM.docx]

**Supplement 2: List of publications included in the review.**

| Aboueid S, Liu RH, Desta BD, Chaurasia A, Ebrahim S. The use of artificially intelligent self-diagnosing digital platforms by the general public: scoping review. *JMIR Med Inform*. 2019; 7(2):e1344: 1-10. doi: 10.2196/13445. |
| --- |
| Albrecht U-V (ed.). Chancen und Risiken von Gesundheits-Apps (CHARISMHA). Hannover 2016. |
| Chambers D, Cantrell AJ, Johnson M, Preston L, Baxter SK, Booth A, et al. Digital and online symptom checkers and health assessment/triage services for urgent health problems: systematic review. *BMJ Open* 2019; 9:e027743: 1-13. doi: 10.1136/bmjopen-2018-027743. |
| Copeland C, Morreale P, Li J. M-Health application interface design for symptom checking: proc. of the 10th Intl. Conf. on e-Health. Madrid 2018: 210-214. |
| Dunn AG. Will online symptom checkers improve health care in Australia? *Med J Aust.* 2020; 212(11): 512-513. doi: 10.5694/mja2.50621. |
| Fiske A, Buyx A, Prainsack B. The double-edged sword of digital self-care: Physician perspectives from Northern Germany. *Social Science & Medicine.* 2020; 260: 1-10. doi: 10.1016/J.SOCSCIMED.2020.113174. |
| Fraser H, Coiera E, Wong D. Safety of patient-facing digital symptom checkers: correspondence. *The Lancet.* 2018; 392(10161): 2263-2264. doi: 10.1016/S0140-6736(18)32819-8. |
| Hageman MGJS, Anderson J, Blok R, Bossen JKL, Ring D. Internet self-diagnosis in hand surgery. *HAND.* 2015; 10: 565-569. doi: 10.1007/s11552-014-9707-x. |
| Herzog C. Technological opacity of machine learning in healthcare. In: Proceedings of the Weizenbaum Conference 2019: "Challenges of Digital Inequality – Digital Education, Digital Work, Digital Life". Berlin 2019: 1-9. doi: 10.34669/wi.cp/2.7. |
| Hill MG, Sim M, Mills B. The quality of diagnosis and triage advice provided by free online symptom checkers and apps in Australia. *Med J Aust.* 2020; 212(11): 514-519. doi: 10.5694/mja2.50600. |
| **Iacobucci G. Row over Babylon’s chatbot shows lack of regulation.** *BMJ.* 2020; 368:m815. doi: 10.1136/bmj.m815. |
| Jimison HB, Sher PP, Jimison JJB. Decision support for patients. In: Berner ES (ed). Clinical Decision Support Systems. Health Informatics. New York 2007: 249-261. doi: 10.1007/978-0-387-38319-4_11. |
| Jutel A, Lupton D. Digitizing diagnosis: a review of mobile applications in the diagnostic process. *Diagnosis*. 2015; 2(2): 89-96. doi: 10.1515/dx-2014-0068. |
| Kao C-K, Liebovitz DM. Consumer mobile health apps: current state, barriers, and future directions. *PM R*. 2017; 9(5S): 106-S115. doi: 10.1016/j.pmrj.2017.02.018. |
| Kramer U. Selbstbestimmter Umgang mit Gesundheits-Apps? Über welche Kompetenzen müssen Verbraucher*innen verfügen? *HiBiFo.* 2017; 2: 16-30. doi: 10.3224/hibifo.v6i2.02. |
| Kuhn S, Jungmann SM, Jungmann F. Künstliche Intelligenz für Ärzte und Patienten „Googeln“ war gestern. *Deutsches Ärzteblatt.* 2018; 115(26): 1262-1266. |
| Kujala S, Hörhammer I, Hänninen-Ervasti R, Heponiemi T. Health professionals' experiences of the benefits and challenges of online symptom checkers. *Stud Health Technol Inform*. 2020; 16(270): 966-970. doi: 10.3233/SHTI200305. |
| Lanseng E, Andreassen TW. Electronic healthcare: a study of people's readiness and attitude toward performing self-diagnosis. *International Journal of Service Industry Management.* 2007; 18(4): 394-417. doi: 10.1108/09564230710778155. |
| Loh E. Medicine and the rise of the robots: a qualitative review of recent advances of artificial intelligence in health. *BMJ Leader.* 2018; 2: 59-63. doi: 10.1136/leader-2018-000071. |
| Luger T, Houston TK, Suls J. Older adult experience of online diagnosis: results from a scenario-based think-aloud protocol. *J Med Internet Res*. 2014; 16(1):e16: 1-12. doi: 10.2196/jmir.2924. |
| Lupton D, Jutel A. ‘It's like having a physician in your pocket!’ A critical analysis of self-diagnosis smartphone apps. *Social Science & Medicine.* 2015; 133: 128-135. |
| Marco-Ruiz L, Bønes E, de la Asunción E, Gabarron E, Aviles-Solis JC, Lee E, et al. Combining multivariate statistics and the think-aloud protocol to assess human-computer interaction barriers in symptom checkers. *Journal of Biomedical Informatics*. 2017; 74: 104-122. |
| Merz S, Bruni T, Bondio MG. Diagnose-Apps: Wenig Evidenz. *Deutsches Ärzteblatt*. 2018; 115(12): 522-524. |
| Meyer AND, Giardiana TD, Spitzmueller C, Shahid U, Scott TMT, Singh H. Patient perspectives on the usefulness of an artificial intelligence-assisted symptom checker: cross-sectional survey study. *J Med Internet Res.* 2020; 22(1):e14679: 1-9. doi: 10.2196/1467. |
| Millenson ML, Baldwin JL, Zipperer L, Singh H. Beyond Dr. Google: the evidence on consumer-facing digital tools for diagnosis. *Diagnosis (Berl).* 2018; 5(3): 95-105. doi: 10.1515/dx-2018-0009. |
| Miller S, Gilbert S, Virani V, Wicks P. Patients’ utilization and perception of an artificial intelligence-based symptom assessment and advice technology in a British primary care waiting room: exploratory pilot study. *JMIR Hum Factors.* 2020; 7(3):e1971: 1-10. doi: 10.2196/19713. |
| Morita T, Rahman A, Hasegawa T, Ozaki A, Tanimoto T. The potential possibility of symptom checker. *Int J Health Policy Manag.* 2017; 6(x): 1-2. doi: 10.15171/ijhpm.2017.41. |
| Nijland N, Cranen K, Boer H, van Gemert-Pijnen JE, Seydel ER. Patient use and compliance with medical advice delivered by a web-based triage system in primary care. *J Telemed Telecare.* 2010; 16(1): 8-11. doi: 10.1258/jtt.2009.001004. |
| Powley L, McIlroy G, Simons G, Raza K*.* Are online symptoms checkers useful for patients with inflammatory arthritis? *BMC Musculoskelet Disord.* 2016; 17(362): 1-6. doi: 10.1186/s12891-016-1189-2. |
| Razzaki S, Baker A, Perov Y, Middleton K, Baxter J, Mullarkey, et al. A comparative study of artificial intelligence and human doctors for the purpose of triage and diagnosis. arXiv:1806.10698 [cs.AI]; 2018. |
| Rowland SP, Fitzgerald E, Holme T, Powell J, McGregor A. What is the clinical value of mHealth for patients? *npj Digital Medicine*. 2020; 3(4): 1-6. doi: 10.1038/s41746-019-0206-x. |
| Ryan A, Wilson S. Internet healthcare: do self-diagnosis sites do more harm than good? *Expert Opin Drug Saf.* 2008; 7(3): 227-9. doi: 10.1517/14740338.7.3.227. |
| Semigran HL, Linder JA, Gidengil C, Mehrotra A. Evaluation of symptom checkers for self diagnosis and triage: audit study*. BMJ.* 2015; 351:h3480: 1-9. doi:10.113 6/bmj.h3480. |
| Shen C, Nguyen M, Gregor A, Isaza G, Beattie A. Accuracy of a popular online symptom checker for ophthalmic diagnoses. *JAMA Ophthalmol*. 2019; 137(6): 690-692. doi: 10.1001/jamaophthalmol.2019.0571. |
| Sönnichsen A. Fluch oder Segen? Symptom Checker und Diagnostik-Apps. *KVH-Journal*. 2019; 9: 32-34. |
| Thielscher C, Antes G. Pro&Contra. Der Arzt behält die Deutungshoheit trotz KI. *Deutsches Ärzteblatt.* 2019; 116(1-2): 18-19. |
| Verzantvoort NCM, Teunis T, Verheij TJM, van der Velden AW. Self-triage for acute primary care via a smartphone application: Practical, safe and efficient? *PLoS One*. 2018; 13(6):e0199284. doi: 10.1371/journal.pone.0199284. |
| Wattanapisit A, Teo CH, Wattanapisit S, Teoh E, Woo WJ, Ng CJ. Can mobile health apps replace GPs? A scoping review of comparisons between mobile apps and GP tasks. *BMC Medical Informatics and Decision Making.* 2020; 20(5): 2-11. doi: 10.1186/s12911-019-1016-4. |
| Wyatt JC. Fifty million people use computerised self triage. *BMJ.* 2015; 351:h3727. doi: 10.1136/bmj.h3727. |
